# CircAFF3 modulation of p53–ID2 signaling in the retinal pigment epithelium links inflammation with cell death in dry age-related macular degeneration

**DOI:** 10.3389/fcell.2026.1733888

**Published:** 2026-03-25

**Authors:** Yeongseo Ryu, Dahee Jeong, Jeong Hun Kim, Young-Kook Kim

**Affiliations:** 1 Department of Biochemistry, Chonnam National University Medical School, Hwasun, Jeollanam-do, Republic of Korea; 2 BioMedical Sciences Graduate Program (BMSGP), Chonnam National University Medical School, Hwasun, Jeollanam-do, Republic of Korea; 3 Fight Against Angiogenesis-Related Blindness (FARB) Laboratory, Clinical Research Institute, Seoul National University Hospital, Seoul, Republic of Korea; 4 Department of Biomedical Sciences, Seoul National University College of Medicine, Seoul, Republic of Korea; 5 Department of Ophthalmology, Seoul National University College of Medicine, Seoul, Republic of Korea

**Keywords:** age-related macular degeneration, circAFF3, geographic atrophy, inflammation, retinal pigment epithelium, RNA-sequencing

## Abstract

**Introduction:**

Age-related macular degeneration (AMD) represents a multifactorial disease that is influenced by age, genetic, and environmental factors. AMD is characterized by dysfunction of the retinal pigment epithelium (RPE) resulting from oxidative stress, inflammation, and complement activation. As the disease progresses, the loss of the RPE and photoreceptors leads to geographic atrophy, which is a hallmark of dry AMD. Although research is ongoing, there is currently no established effective treatment for dry AMD. Notably, circular RNAs (circRNAs) have been studied in various diseases; however, the role of circRNAs in eye diseases remains poorly understood. To fill this gap, this study aimed to investigate circRNAs as potential therapeutic targets for dry AMD.

**Methods:**

We identified candidate circRNAs using a laser-induced choroidal neovascularization (CNV) model. The function of circAFF3 was investigated through knockdown experiments in ARPE-19 cells, followed by transcriptomic analysis, pathway enrichment, and functional assays, including qPCR, western blotting, immunofluorescence, monocyte adhesion, and measurements of ROS, iron, lipid peroxidation, and apoptosis. Interaction between circAFF3 and p53 was explored using binding prediction, RNA-binding protein immunoprecipitation, and cycloheximide chase assay.

**Results:**

We found that circAff3 levels were downregulated in RPE samples at day 3 after laser injury. Moreover, silencing circAFF3 induced an inflammatory response in ARPE-19 cells. Based on these results, a subsequent transcriptomic analysis of circAFF3 knockdown in ARPE-19 cells was conducted to further elucidate its function. These analyses showed that genes downregulated by circAFF3 knockdown in ARPE-19 cells were significantly associated with retinal degeneration. Additionally, reduced circAFF3 expression promoted a decrease in ID2 levels, resulting in increased oxidative stress and cell death. Our study further demonstrated that circAFF3 directly interacts with p53, thereby regulating ID2 expression in ARPE-19 cells.

**Conclusion:**

Collectively, our study reveals that circAFF3 plays a crucial role in RPE dysfunction by modulating a circAFF3/p53/ID2 pathway, suggesting that circAFF3 can serve as a key regulator with therapeutic potential in dry AMD.

## Introduction

Age-related macular degeneration (AMD) is a progressive retinal disease that represents a leading cause of irreversible vision loss in individuals aged ≥60 years, accounting for 8.7% of all blindness worldwide (Guymer and Campbell, 2023; Wong et al., 2014). Thus, as society continues to age, the global burden of vision impairment due to AMD is expected to increase from 8.06 million cases in 2021 to about 21.34 million cases by 2050 (Collaborators, 2025). AMD primarily affects the macula, the central part of the retina responsible for high-acuity tasks such as reading, driving, and facial recognition, underscoring the association of AMD with physical and mental wellbeing (Guymer and Campbell, 2023). Clinically, AMD is classified into two forms: non-vascular or “dry” and neovascular or “wet” (Mitchell et al., 2018). The dry form typically progresses slowly, beginning with the accumulation of extracellular deposits called drusen between the retinal pigment epithelium (RPE) and Bruch’s membrane (BrM) (Schultz et al., 2021). The advanced stage of dry AMD, termed geographic atrophy (GA), leads to irreversible loss of retinal structures, including the photoreceptors, RPE, BrM, and choriocapillaris (Fleckenstein et al., 2018). In contrast, wet AMD is characterized by the abnormal growth of fragile choroidal vessels into the subretinal space; these vessels are prone to leakage and hemorrhage, resulting in rapid and severe vision loss (Lim et al., 2012). Currently, anti-vascular endothelial growth factor (VEGF) therapies, such as ranibizumab, aflibercept, and off-label bevacizumab, represent highly effective methods for managing wet AMD (Mitchell et al., 2018). Meanwhile, despite dry AMD accounting for approximately 85%–90% of all cases and severely impacting vision, effective treatments remain extremely limited (Schultz et al., 2021).

The development of effective treatments for dry AMD requires an understanding of the pathophysiological pathways leading to RPE degeneration (Datta et al., 2017). The RPE plays a vital role in maintaining visual homeostasis by forming the blood–retinal barrier, regulating the visual cycle, performing daily phagocytosis of photoreceptor outer segment (POS) debris, and reducing oxidative stress (Yang et al., 2021). Importantly, aging increases reactive oxygen species (ROS) production in the RPE under continuous light exposure, making the cells more susceptible to oxidative stress, which damages proteins, lipids, and DNA, promoting structural alterations. These oxidative modifications generate oxidation-specific epitopes (OSEs), which can activate proinflammatory signaling pathways, particularly the nuclear factor kappa-light-chain-enhancer of activated B cells (NF-κB) pathway (Datta et al., 2017; Tan et al., 2020; Almalki and Almujri, 2024). NF-κB activation promotes the production of proinflammatory cytokines, including IL-1β, IL-6, IL-8, IL-12, IL-17, TNF-α, and IFN-γ; chemokines, such as MCP-1; and adhesion molecules, e.g., ICAM-1 and VCAM-1. Excessive NF-κB activation leads to the sustained overproduction of these mediators, resulting in chronic inflammation, immune cell infiltration, and retinal tissue damage (Almalki and Almujri, 2024). In the context of dry AMD, a prolonged period of oxidative stress and NF-κB-driven inflammation is believed to accelerate RPE degeneration and promote GA (Almalki and Almujri, 2024; Kauppinen et al., 2016; Rajanala et al., 2023). Thus, investigating these inflammatory mechanisms may provide a foundation for effective therapeutic strategies.

Circular RNAs (circRNAs), generated from precursor mRNAs, are a new type of non-coding RNA with a covalently closed circular form. CircRNAs are formed through the back-splicing process, in which the spliceosomal machinery joins the 5′splice site in the downstream intron to the 3′splice site in the upstream intron (Zhou et al., 2020). Since circRNAs comprise a circular structure, these RNAs are highly stable compared to linear RNAs, with an average half-life of over 48 h (Jeck and Sharpless, 2014). Moreover, the biogenesis of circRNAs can be facilitated by cis-elements, such as intronic complementary sequences (ICSs), and trans-factors, including RNA-binding proteins (RBPs). Notably, while the biogenesis of circRNAs occurs in the nucleus, most exonic circRNAs (ecircRNAs) are exported to the cytoplasm via the nuclear export system. Increasing evidence highlights that circRNAs exert diverse cellular functions, including acting as microRNA (miRNA) sponges, interacting with proteins, regulating transcription, and, in some cases, encoding proteins such as circMAP3K4 (Misir et al., 2022; Duan et al., 2022). Recently, circRNAs have been extensively studied in cancer, heart disease, and neurological disorders, underscoring their importance in human pathology (Wang et al., 2022b; Ju et al., 2023; Song et al., 2022). Nevertheless, the roles of circRNAs in retinal degeneration, particularly in dry AMD, remain largely unknown.

In our study, to identify circRNAs potentially involved in dry AMD pathogenesis, we used a laser-induced choroidal neovascularization (CNV) model. This CNV model reproduces key features of wet AMD by disrupting BrM and promoting subretinal blood vessel growth (Lambert et al., 2013). We analyzed circRNA expression at an early stage in this model and identified circAFF3 as a candidate. Subsequently, we confirmed that depleting circAFF3 in ARPE-19 cells triggers inflammatory responses. Additionally, circAFF3 was shown to regulate oxidative stress and cell death through the circAFF3/p53/*ID2* axis. Overall, we anticipate that our findings may provide insights for circRNA-based therapeutic strategies in dry AMD.

## Materials and methods

### Animal care

All animal procedures were conducted in strict accordance with the ARVO Statement for the Use of Animals in Ophthalmic and Vision Research, the guidelines of the College of Veterinary Medicine, and protocols approved by the Institutional Animal Care and Use Committee at Seoul National University (Permit Number: SNU-230731-4-4). C57BL/6 (Stock no. 000664) mice were obtained from the Jackson Laboratory (Bar Harbor, Maine, United States). Eight-week-old male C57BL/6J mice were used in all experiments and maintained under pathogen-free conditions with a 12-h light-dark cycle and *ad libitum* access to food and water in approved cages.

### Laser-induced CNV model

Mice were anesthetized prior to laser treatment, and their pupils were dilated with a solution containing topical phenylephrine (0.5%) and tropicamide (0.5%). Laser photocoagulation was performed using an indirect headset delivery system (Ilooda, Suwon, Gyeonggi-do, KR) and a laser device (Ilooda) with the following parameters: wavelength 810 nm, spot size 200 μm, power 1 W, and irradiation time 100 ms. Laser irradiation was applied 3–4 times around the optic disc, and only areas where bubbles formed without vitreous hemorrhage were included in the analysis. The CNV-positive areas in all laser-irradiated sites (four per eye) were quantitatively measured using the LAS X system (TCS SP8; Leica Microsystems, Wetzlar, DE). CNV volume was quantified using Imaris software (Oxford Instruments, Abingdon-on-Thames, United Kingdom).

To isolate the RPE, mice were euthanized via carbon dioxide overdose. Eyes were opened just posterior to the limbus using Vannas scissors, and the anterior segment (cornea, iris, and lens) was removed. The retina was carefully dissected to expose the RPE-choroid-sclera (RCS) complex. The posterior eyecups containing the RCS complex were transferred to small Petri dish and flash-frozen in liquid nitrogen. Subsequently, 200 μL of PBS was injected into the posterior eyecup using a pipette with a 200-μL tip. This vigorous injection process was repeated 20 times with the same PBS solution. The resulting PBS suspension, containing brown clumps of released material presumed to be RPE cells, was collected into 1.5-mL microcentrifuge tubes.

### Cell culture

The human RPE cell line ARPE-19 was obtained from the American Type Culture Collection (ATCC) and maintained in Dulbecco’s Modified Eagle’s Medium/Nutrient Mixture F-12 (DMEM/F-12; WELGENE, LM002-05) supplemented with 10% fetal bovine serum (FBS; WELGENE, S001-01) and 1% antibiotic/antimycotic solution (WELGENE, LS203-01). To establish the *in vitro* model of dry AMD, ARPE-19 cells were exposed to 10 mM NaIO_3_ (Sigma-Aldrich, 71702). The human monocyte cell line THP-1 was obtained from the Korean Cell Line Bank and cultured in RPMI 1640 (WELGENE, LM001-51) supplemented with 10% FBS, 1% antibiotic/antimycotic solution, and 0.05 mM 2-mercaptoethanol (Gibco, 21985-023). Human retinal microvascular endothelial cells (HRMECs) were purchased from Cell Systems and cultured in EGM®-2 Endothelial Cell Growth Medium-2 BulletKit® (Lonza, CC-3162). All cells were incubated at 37 °C in a humidified atmosphere containing 5% CO_2_.

### RNA isolation and PCR

Total RNA was extracted using TRIzol reagent (Invitrogen), and residual DNA was removed by treatment with recombinant DNase I (Takara), according to each manufacturer’s instructions. RNA purity and concentration were measured on a NanoPhotometer (IMPLEN), and RNA integrity was confirmed by electrophoresis on a 1% agarose gel. Equal RNA concentrations were reverse-transcribed using random hexamers and RevertAid reverse transcriptase (Thermo Fisher Scientific). For semi-quantitative PCR (semi-qPCR), complementary DNA (cDNA) was amplified using Phusion High-Fidelity DNA Polymerase (Thermo Fisher Scientific) with gene-specific primers in the Mastercycler Nexus X2 (Eppendorf). The PCR products were separated on a 2% agarose gel, and band intensity was quantified using ImageJ (Version 1.54d). The gene expression levels were normalized to those of *glyceraldehyde 3-phosphate dehydrogenase* (*GAPDH*). Quantitative real-time PCR (qPCR) was performed using Power SYBR Green PCR Master Mix (Applied Biosystems) in the Rotor-Gene Q real-time PCR system (QIAGEN). Relative expression levels were calculated using the 2^−ΔCt^ method, with *GAPDH* as the loading control. The primer sequences used in the PCR are listed in Supplementary Table 1.

### RNA sequencing (RNA-seq) analysis

Total RNA was isolated from the RPE tissues of the laser-induced CNV model mice to identify differentially expressed circRNAs and mRNAs. DNase I was used to eliminate residual DNA according to the manufacturer’s protocol. Subsequently, RNA quality was verified using the Agilent 2,100 Bioanalyzer (Agilent). The Ribo-Zero Gold rRNA Removal kit (Illumina) was employed to remove the ribosomal RNA, and the TruSeq Stranded Total RNA kit (Illumina) was then utilized to construct the RNA sequencing library. Sequencing was performed on a HiSeq 2,500 platform (Illumina) in paired-end mode, using 100 cycles per read. After sequencing, low-quality reads were filtered out using Trimmomatic (Bolger et al., 2014). The circRNA expression levels were determined using the STAR aligner, a tool for mapping RNA sequences (Dobin et al., 2013), and the DCC algorithm, which detects circular RNAs (Cheng et al., 2016). The circRNA counts from the triplicate samples were combined, and the expression levels were normalized to the total circRNA counts in each sample.

To screen for changes in the mRNAs following circAFF3 depletion, total RNA was extracted from mock-transfected and circAFF3-depleted ARPE-19 cells using TRIzol reagent. The TruSeq Stranded mRNA kit (Illumina) was utilized in the construction of the RNA sequencing libraries to capture poly-adenine-tailed mRNAs. Sequencing data quality was verified using FastQC, and low-quality reads were trimmed using the Trimmomatic algorithm. Two approaches were applied and intersected to analyze the expression of specific mRNAs. First, the STAR aligner was used to map sequencing reads to the human genome (hg19), and fragments per kilobase of transcript per million mapped reads (FPKM) were determined using Cuffnorm (Dobin et al., 2013; Trapnell et al., 2012). Second, transcripts from the RNA-seq analysis were quantified using a Salmon quantifier, and gene expression changes were assessed using edgeR (Patro et al., 2017; Robinson et al., 2010). Genes with an average FPKM value of 1 or less were excluded from the analysis, and genes with a *p*-value of less than 0.05 were selected as analysis targets.

### RNase R treatment and sanger sequencing

Total RNA extracted from ARPE-19 cells was incubated with RNase R (Biosearch Technologies) and 10x RNase R reaction buffer to degrade linear RNA and confirm the circularity of the circRNAs. In the control group, only the reaction buffer was added. The mixtures were incubated at 37 °C for 10 min, followed by the inactivation of RNase R at 95 °C for 3 min. Expression levels of circRNAs and mRNAs were assessed using semi-qPCR. PCR products were excised under an ultraviolet (UV) transilluminator, and the sequences flanking the back-splicing junctions of the circRNAs were verified by Sanger sequencing (Solgent).

### Subcellular fractionation

To separate the cytoplasmic and nuclear fractions from the ARPE-19 cells and HUVECs, the cells were washed twice with phosphate-buffered saline (PBS), scraped, and then transferred into microcentrifuge tubes. After centrifugation, the cell pellets were lysed in cold buffer A (10 mM HEPES buffer (pH 7.9), 10 mM KCl, 1 mM DTT, and 0.1 mM EDTA) and incubated on ice for 25 min. Then, 10% Nonidet P-40 (NP-40) was added, mixed, and the lysates were incubated on ice for 2 min. Following centrifugation, the cytoplasmic RNA in the supernatant was extracted using TRIzol LS reagent (Invitrogen), and the cytoplasmic proteins were collected in a buffer containing protease and phosphatase inhibitors. The nuclear pellets were washed three times with K100 buffer D (20 mM Tris (pH 8.0), 100 mM KCl, and 0.2 mM EDTA), and the nuclear RNA was extracted using TRIzol reagent. For nuclear protein extraction, the pellet was resuspended in lysis buffer containing protease and phosphatase inhibitors and sonicated using a Bioruptor (Diagenode). Precursor *GAPDH* (pre-*GAPDH*) was used as a nuclear marker, while *GAPDH* and *ACTB* were employed as cytoplasmic markers. Histone H3 and Lamin B1 were utilized as nuclear protein markers, while GAPDH was used as a cytoplasmic protein marker.

### *In situ* hybridization

The spatial distribution of circAFF3 was confirmed in ARPE-19 cells using the BaseScope™ Detection Reagent Kit v2-RED (Advanced Cell Diagnostics [ACD], 323900) according to the manufacturer’s protocol. The circAFF3 probe targeting its splicing junctions was designed and synthesized from ACD. For quality control of the *in situ* hybridization (ISH) procedure, control probes targeting *PPIB* and *dapB* were used as positive and negative controls, respectively. Briefly, slides were dehydrated and rehydrated using a series of ethanol dilutions (50%, 70%, and 100%). After washing with PBS, the slides were incubated in RNAscope hydrogen peroxide solution at room temperature for 10 min, followed by washing with PBS and treated with 1:15 diluted protease III solution at room temperature for 10 min. After washing twice with PBS, each appropriate probe was hybridized to each gene in a HybEz™ oven at 40 °C for 2 h. Then, the signal from each gene was amplified using AMP 1-8 reagents. Finally, to detect circAFF3 chromogenic signal, slides were incubated with Basescope™ Fast RED for 10 min, and counterstained with 50% Gill’s hematoxylin and 0.02% ammonia water for 2 min at room temperature for nuclear staining. After mounting the slides with VectaMount (Vector Laboratories), chromogenic images were visualized using an Eclipse Ts2 fluorescence microscope (Nikon).

### Constructions of siRNA and plasmid

The siRNAs targeting circAFF3 and *MDM2* were designed using the siDESIGN Center from Horizon Discovery (https://horizondiscovery.com/en/products/tools/siDESIGN-Center) and i-Score Designer (https://www.med.nagoya-u.ac.jp/neurogenetics/i_Score/i_score.html). The synthesized siRNAs, along with the AccuTargetTM negative control siRNA, were obtained from Bioneer. The sequences of the used siRNAs are listed in Supplementary Table 2.

Cosmo Genetech constructed the plasmid for circAFF3 overexpression. Primers were designed to amplify sequences for circAFF3 and restriction sites (EcoRV and SacII). The pcDNA 3.1 (+) Zkscan1 MCS exon vector (Addgene, plasmid #69901), provided by Jeremy Wilusz, served as the backbone to facilitate the formation of the circRNA. Both the vector and the insert were digested using EcoRV and SacII restriction enzymes, then ligated using the EZ-Fusion Cloning kit (Enzynomics), according to the manufacturer’s instructions. The primer sequences used for the PCR are listed in Supplementary Table 3.

### Transfection

To perform the circAFF3 and *MDM2* knockdown, 1.5 × 10^4^ cells per cm^2^ were seeded in multi-well plates. On the following day, siRNAs targeting circAFF3 and *MDM2* were transfected into ARPE-19 cells using Lipofectamine™ 3,000 and RNAiMAX (Invitrogen), respectively, according to the manufacturer’s instructions. The siRNA was used at a final concentration of 30 nM. For the circRNA overexpression assay, 2 × 10^5^ ARPE-19 cells were seeded into each well of a 6-well plate. The next day, 1 μg of plasmid was transfected into ARPE-19 cells using Lipofectamine™ 3,000. Then, 6 h after transfection, the medium was replaced with DMEM/F-12 containing 10% FBS, and cells were incubated for an additional 42 or 66 h. To perform a reverse transfection, cells were seeded directly with a transfection mixture. The following day, the medium was replaced with DMEM/F-12 containing 10% FBS, and the cells were cultured for an additional 24 h.

### Western blot analysis

Proteins were extracted from ARPE-19 cells using ice-cold radio-immunoprecipitation assay (RIPA) buffer (Biosesang) supplemented with protease and phosphatase inhibitors (GenDEPOT). Lysates were incubated on ice for 30 min and centrifuged at 14,000 × g for 15 min. Protein concentrations were quantified using the Pierce BCA Protein Assay kit (Thermo Fisher Scientific). Equal amounts (20–30 µg) of the protein lysates were separated using sodium dodecyl sulfate–polyacrylamide gel electrophoresis (SDS–PAGE). Proteins were transferred to polyvinylidene difluoride (PVDF) membranes (Merck Millipore) that had been activated using methanol (Merck). The membranes were blocked with 5% bovine serum albumin (BSA) (GenDEPOT) at room temperature for 1 h and then incubated overnight at 4 °C with primary antibodies against NF-κB p65 (Cell Signaling Technology, 8242s), phosphorylated NF-κB p65 (Cell Signaling Technology, 3033s), ICAM-1 (Cell Signaling Technology, 4915s), cleaved PARP (Cell Signaling Technology, 9541s), BAX (Cell Signaling Technology, 2772s), p53 (Santa Cruz, sc-126), MDM2 (Santa Cruz, sc-13161), Histone H3 (Cell Signaling Technology, 4499s), Lamin B1 (Abcam, ab16048), and GAPDH (Santa Cruz, sc-32233). The following day, each membrane was washed three times with 1× Tris-buffered saline and 0.1% Tween 20 (TBS-T) and incubated with a horseradish peroxidase-conjugated secondary antibody (1:5,000) at room temperature for 1 h. Protein bands were visualized using Immobilon Western Chemiluminescent HRP Substrate solution (Millipore) and Fusion Solo (Vilber). Band intensities were quantified using ImageJ (Version 1.54d). Protein expression levels were normalized to GAPDH, and phosphorylated protein levels were normalized to the total protein.

### Immunofluorescence staining

ARPE-19 cells were reverse-transfected, fixed with 4% paraformaldehyde (PFA) for 15 min, and permeabilized with 0.2% Triton X-100 at room temperature for 5 min. Cells were then blocked with 2% BSA at room temperature for 1 h. Primary antibodies against ICAM-1 and p53 were added and incubated overnight at 4 °C. Subsequently, cells were incubated with fluorophore-conjugated secondary antibodies at room temperature for 1 h. Nuclei were stained with 0.5 μg/mL 4′,6-diamidino-2-phenylindole (DAPI) at room temperature for 5 min. Fluorescent images were acquired using a Zeiss LSM 900 confocal laser microscope (Carl Zeiss). Images were visualized and processed using ZEN 3.1 (Blue edition, Carl Zeiss), and fluorescence intensity was quantified using ImageJ software (Version 1.54d).

### Monocyte adhesion assays

First, 5 × 10^5^ THP-1 cells were labeled with 5 μM calcein AM (Invitrogen) at 37 °C for 30 min. In parallel, circAFF3-depleted ARPE-19 cells were stained with 1 μM CellTracker™ Red (Invitrogen) at 37 °C for 30 min. Labeled THP-1 cells were then seeded into wells containing the stained ARPE-19 cells. After 2 h of co-culture, non-adherent cells were removed by washing three times with PBS. Five random fields for each insert were captured using the Eclipse Ts2 fluorescence microscope (Nikon), and the number of attached THP-1 cells was counted using ImageJ (Version 1.54d).

### Bioinformatics analysis

Hallmark gene set enrichment analysis (GSEA) was conducted to examine the biological processes associated with circAFF3 knockdown using two approaches. First, protein-coding genes with a log_2_-transformed count per million (CPM) value of ≥1 were analyzed using GSEA with the MSigDB hallmark gene sets (Subramanian et al., 2005). Second, significantly expressed genes with a *p-*value <0.05 and a Log_2_(CPM) value ≥4 were selected and directly examined using the MSigDB web-based hallmark analysis tool (https://www.gsea-msigdb.org/). The most enriched hallmark gene sets were assessed based on their normalized enrichment score (NES) and false discovery rate (FDR) *q*-value.

Potential transcription factors regulating *ID2* and *AFF3* were identified using the JASPAR database (Rauluseviciute et al., 2024). To prioritize candidates, transcription factors were evaluated based on their JASPAR scores, which represent the similarity between genomic sequences and transcription factor binding motifs. Only those with scores ≥400, corresponding to *p* ≤ 0.0001, were considered. Additionally, the Human–Mouse–Rat (HMR) Conserved track in the UCSC Genome Browser was utilized to evaluate the evolutionary conservation of the predicted binding sites (https://www.gene-regulation.com/) (Choy et al., 2010). Specifically, those with a Z-score ≥1.64, corresponding to a *p*-value of 0.05, were included for further analysis.

To investigate the disease and pathway involvements of downregulated genes after circAFF3 knockdown in ARPE-19 cells, we performed functional enrichment analysis with EnrichR (https://maayanlab.cloud/Enrichr/). Disease relevance was assessed using the Jensen Disease Curated 2025 database, and pathway enrichment was evaluated using the WikiPathways 2024 Human database. The top 10 terms were ranked according to–log_2_ (*p*-value).

RNA–protein interactions between the circAFF3 and p53 protein were predicted using RPISeq, a tool that estimates binding probabilities based on random forest (RF) and support vector machine (SVM) classifiers (http://pridb.gdcb.iastate.edu/RPISeq/).

### ROS measurement

Intracellular reactive oxygen species (ROS) were measured using 2′,7′-dichlorofluorescein diacetate (DCFH-DA; Sigma-Aldrich). A total of 1.5 × 10^5^ ARPE-19 cells were seeded in 6-well plates, and the next day, circAFF3 was knocked down for 48 and 72 h. The medium was then replaced with DMEM/F-12 medium containing 10 μM DCFH-DA, and the cells were incubated at 37 °C for 30 min. After the reaction was completed, the cells were washed with PBS, and 1 mL of DMEM/F-12 medium containing 10% FBS was added to each well. Fluorescence images were acquired using an Eclipse Ts2 fluorescence microscope (Nikon). Then, the medium was removed, and 600 μL of RIPA buffer was added to each well. Cells were collected using a scraper and transferred to microcentrifuge tubes. After incubating on ice for 10 min, the samples were centrifuged at 13,000 × g and 4 °C for 15 min, and 100 μL of the supernatant was transferred to a black 96-well plate. The fluorescence intensity was measured using a SpectraMax M2 microplate reader (Molecular Devices) at an excitation wavelength of 485 nm and an emission wavelength of 530 nm.

### Cell viability assay

To assess the effect of circAFF3 knockdown on ARPE-19 cell proliferation, 1.5 × 10^5^ ARPE-19 cells were seeded into 6-well plates and cultured for 48 h. After knockdown, EZ-Cytox reagent (DoGEN) was added to each well at 10% of the culture medium volume and incubated at 37 °C for 2 h. Cell viability was then measured according to the manufacturer’s instructions. Absorbance was measured at 450 nm, with 650 nm used as a reference, using a microplate reader (BioTek).

### TUNEL assay

To detect nuclear fragmented DNA, a hallmark of apoptosis, in ARPE-19 cells following circAFF3 knockdown, we used the DeadEnd™ Fluorometric TUNEL (terminal deoxynucleotidyl transferase-mediated dUTP nick-end labeling) System (Promega) according to the manufacturer’s instructions. First, precleaned 18 mm glass slides were coated with 0.1 mg/mL poly-D-lysine (Gibco). A reverse-transfection was then performed on 8 × 10^4^ ARPE-19 cells for circAFF3 knockdown. After incubating for 48 h, the cells were fixed with 4% PFA (GeneAll) at 4 °C for 25 min and washed twice with PBS. For permeabilization, the slides were immersed in 0.2% Triton X-100 (Sigma-Aldrich) at room temperature for 5 min, followed by two washes with PBS. Next, we equilibrated the cells with Equilibration Buffer at room temperature for 5 min to stabilize the intracellular environment. Subsequently, rTdT incubation buffer was added to label the fragmented DNA, and the reaction was terminated by immersing the cells in 2× SSC. Cell nuclei were counterstained and mounted using ProLong™ Gold Antifade Mountant with DAPI (Thermo Fisher Scientific). Images were captured using an Eclipse Ts2 fluorescence microscope (Nikon). Apoptotic cells were quantified using the cell counter plugin in ImageJ (Version 1.54d). The percentage of apoptotic cells was determined using the following formula (number of TUNEL-positive cells/total number of nuclei) × 100.

### Measurement of ferroptosis markers

To detect the intracellular ferrous iron content, circAFF3 was knocked down in ARPE-19 cells for 48 h. Cells were subjected to circAFF3 knockdown for 72 h. For NF-κB inhibition experiments, TPCA-1 (5 μM, Tocris) was added 6 h after transfection and maintained for an additional 66 h. After washing the cells with serum-free DMEM/F-12 medium three times, the cells were stained with 1 µM FerroOrange (Dojindo) according to the manufacturer’s instructions. The cells were then incubated at 37 °C for 30 min and immediately observed using an Eclipse Ts2 fluorescence microscope (Nikon). Fluorescence intensity was measured using ImageJ software (Version 1.54d).

The BODIPY™ 581/591 C11 (Invitrogen) was used to measure lipid peroxidation in ARPE-19 cells. After 72 h of circAFF3 knockdown, cells were incubated with 5 µM of the C11-BODIPY probe at 37 °C for 30 min, washed with serum-free DMEM/F-12 medium, and visualized using an Eclipse Ts2 fluorescence microscope (Nikon). Fluorescence was assessed by a SpectraMax M2 microplate reader (Molecular Devices) at excitation/emission wavelengths of 581/591 nm, respectively, for the reduced form and at 488/510 nm for the oxidized form. The ratio of oxidized to reduced fluorescence was then calculated.

### RNA–binding protein immunoprecipitation

The circAFF3–p53 interaction was analyzed by RNA–binding protein immunoprecipitation (RIP) using the Magna RIP™ RNA–Binding Protein Immunoprecipitation kit (Millipore) according to the manufacturer’s instructions. Approximately 1 × 10^7^ ARPE-19 cells were lysed in RIP lysis buffer on ice for 5 min. Magnetic beads were preincubated with a p53 antibody or normal mouse IgG as a negative control, then mixed with RIP buffer. Subsequently, 100 µL of cell lysate was added and incubated at 4 °C with gentle rotation overnight. After incubation, the tubes were briefly centrifuged and then placed on a magnetic separator to remove supernatant. Proteinase K treatment was performed at 55 °C for 1 h to remove proteins. RNA was then extracted using phenol–chloroform (Fisher BioReagents™, BP1754I), followed by chloroform, and finally precipitated with ethanol. The extracted RNA was reverse-transcribed, and the co-precipitated RNA was assessed by semi-qPCR.

### Cycloheximide chase assay

Cycloheximide (CHX; Sigma-Aldrich) was used to determine the half-life of the protein in ARPE-19 cells following circAFF3 knockdown. After incubating for 48 h, the cells in each group were treated with 100 μg/mL CHX for varying durations, ranging from 0 to 180 min. Cells were harvested at 0, 60, 90, 120, and 180 min. Proteins were extracted and analyzed for p53 expression using Western blotting.

### Statistical analysis

All data are presented as the mean ± SD. An unpaired two-tailed test with Welch’s correction was performed to compare control and experimental samples in GraphPad Prism 8. Results were considered statistically significant at *p < 0.05, **p < 0.01, ***p < 0.005, and ****p < 0.001.

## Results

### Identification of circRNAs related to dry AMD

**FIGURE 1:**
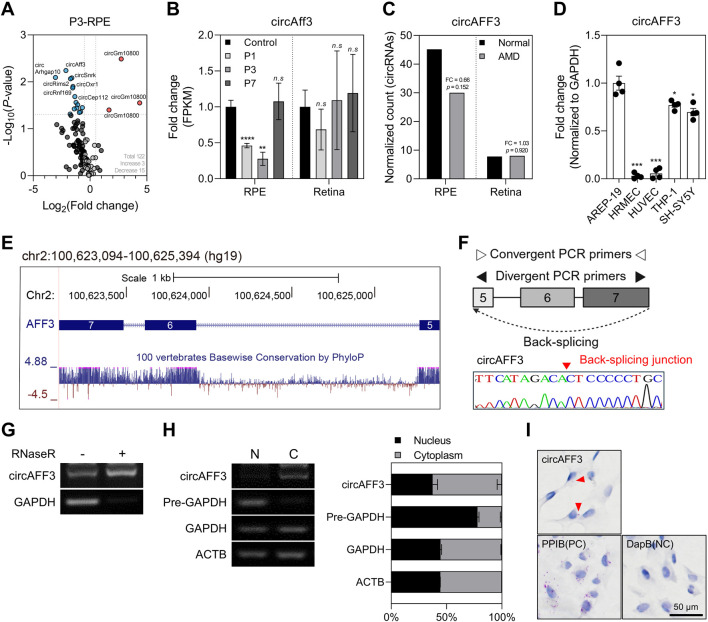
Characterization of circAFF3 in relation to dry AMD in the retinal pigment epithelium (RPE). **(A)** Differentially expressed circRNAs in the RPE samples at day 3 after the laser-induced choroidal neovascularization (CNV). Genes with a value of *p* ≤ 0.05 and |log_2_ (fold change)| ≥ 0.5 are indicated as red (upregulated) and blue (downregulated) dots. **(B)** Changes in circAFF3 circAfff3 expression in the RPE and retina after laser treatment compared with the untreated group (n = 3). **(C)** Normalized read counts of circAFF3 in the RPE and retina of normal and AMD patients, obtained from dataset GSE99248. **(D)** Semi-quantitative RT-PCR (semi-qPCR) analysis of circAFF3 expression in five different cell lines (n = 4). Expression levels of circAFF3 were normalized to those of GAPDH, and all groups were compared to ARPE-19 cells. ARPE-19: human retinal pigment epithelial cells; HRMEC: human retinal microvascular endothelial cells; HUVEC: human umbilical vein endothelial cells; THP-1: human monocyte cells; SH-SY5Y: human neuroblastoma cells. **(E)** Genomic information and evolutionary conservation of the circAFF3 locus as displayed in the UCSC Genome Browser (hg19). **(F)** Schematic of the circAFF3 amplification strategy using divergent PCR primers. The back-splice junction of circAFF3 was validated by Sanger sequencing of the PCR products. **(G)** Validation of the circular structure of circAFF3 by RNase R treatment using semi-qPCR (n = 3). Expression levels in the RNase R-treated groups were compared with those in the mock-treated controls. **(H)** Distribution of circAFF3 in the nuclear (N) and cytoplasmic **(C)** fractions of ARPE-19 cells, as determined by semi-qPCR (n = 3). Pre-GAPDH expression was used as a nuclear marker, while GAPDH and ACTB were used as cytoplasmic markers. Values represent the percentage in each fraction relative to the total amount. Data are shown as the mean ± standard deviation (SD). An unpaired two-tailed t-test with Welch’s correction was used for statistical analysis (ns, not significant; *, *p* < 0.05; **, *p* < 0.01; ***, *p* < 0.005; ****, *p* < 0.001). **(I)** *In situ* hybridization of circAFF3 in ARPE-19 cells. circAFF3 was detected using a specific probe to target its back-splice junction. Probes targeting *PPIB* and bacterial *dapB* were used as positive and negative controls, respectively. The red arrow points to stained circAFF3. circAFF3 is shown as a red dot, and the nucleus is shown in blue.

To screen candidate circRNAs potentially involved in dry AMD pathogenesis, we analyzed RNA-seq data from RPE samples collected at days 1, 3, and 7 following laser injury in a laser-induced CNV model. Previous studies have reported that inflammatory macrophage and monocyte subsets infiltrate the lesion site and reach maximum levels at day 3 after laser photocoagulation (Lambert et al., 2013; Tan et al., 2015). Therefore, we focused on the transcriptomic changes at day 3, corresponding to the peak of the inflammatory response implicated in the mechanisms of dry AMD. RNA-seq analysis revealed a total of 122 circRNAs expressed in the RPE samples, with 18 circRNAs showing significant alterations (3 upregulated and 15 downregulated genes) (Figure 1A). To select the appropriate candidate circRNAs, we applied the following criteria: accurate alignment of back-splicing junctions with annotated exon boundaries, conservation in the human genome, and limited prior research. Notably, circAFF3 has been reported to be abundantly expressed in B cells; however, the function of circAFF3 has yet to be fully characterized (Gaffo et al., 2019). The role of *AFF3*, the host gene of circAFF3, in retinal research has also been poorly studied. Based on these criteria, we selected circAFF3 for further study. The circAff3 expression was significantly reduced in the RPE samples, but not in the RPE-excluded retina samples, at days 1 and 3 post-laser injury (Figure 1B). Furthermore, to explore whether a similar expression pattern could be observed in human RPE, we analyzed publicly available Gene Expression Omnibus (GEO) datasets derived from RPE and RPE-excluded retina samples of individuals with AMD and healthy controls. Consistent with the mouse data, circAFF3 expression was downregulated in the RPE of AMD samples compared to healthy controls. However, there was no change in expression in the retina samples, where expression was lower than in the RPE cells (Figure 1C). Moreover, circAFF3 was significantly enriched in the ARPE-19 cells compared to other cell types, supporting the previously observed RPE-specific expression pattern (Figure 1D). Thus, we selected circAFF3 for further investigation in ARPE-19 cells to explore its potential role in retinal degeneration.

### Circular structure and cytoplasmic localization of circAFF3

We identified that circAFF3 originates from exons 5 to 7 in the *AFF3* host gene, which is located on chromosome 2 at the q11.2 region in the human genome (Figure 1E). To verify the circular nature of circAFF3 in ARPE-19 cells, we designed divergent primers from exons 5 and 7 to amplify PCR products containing the back-splicing junction (Figure 1F). The PCR products were treated with RNase R, an enzyme that degrades linear RNAs. We found that circAFF3 was resistant to RNase R digestion, whereas the linear RNA GAPDH was degraded, confirming the circular structure of circAFF3 (Figure 1G). Since the functions of circRNAs often depend on the respective cellular compartment (Misir et al., 2022), we performed subcellular fractionation in ARPE-19 cells and HUVECs and *in situ* hybridization in ARPE-19 cells to determine the intracellular distribution of circAFF3. Our result showed that circAFF3 was mainly located in the cytoplasmic fraction (Figures 1H,I; Supplementary Figure S1). These findings suggest that circAFF3 may contribute to the pathogenesis of AMD by regulating cytoplasmic molecular pathways.

### Regulation of inflammatory responses through NF-κB signaling by circAFF3

**FIGURE 2:**
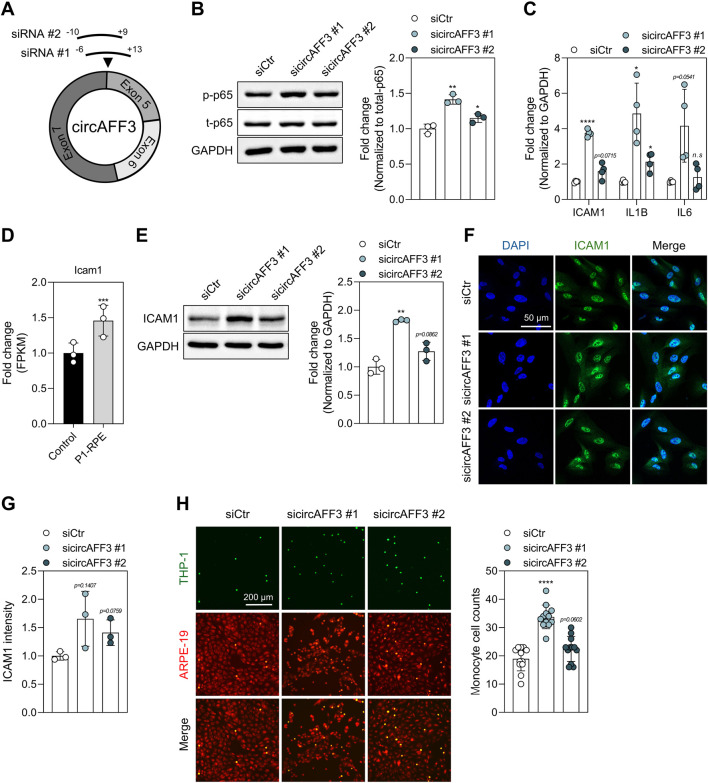
Induction of inflammatory response via p65 signaling in circAFF3-depleted ARPE-19 cells. **(A)** Illustration of the design of the used siRNAs (#1 and #2) for circAFF3 knockdown. The binding sites of each siRNA are indicated by ‘-’ and ‘+’ based on the back-splicing junction (0) represented by the black arrow. **(B)** Western blot analysis of p65 activation in circAFF3-depleted ARPE-19 cells (n = 3). Expression levels of phosphorylated p65 (p-p65) were normalized to those of total p65. **(C)** Quantitative real-time PCR (qPCR) analysis of the expression changes of proinflammatory genes following circAFF3 knockdown in ARPE-19 cells (n = 4). **(D)** Fold change for the fragments per kilobase of exon per million mapped reads (FPKM) of *Icam1* in the RPE at day 1 post-laser irradiation (n = 3). The laser-treated group was compared to the untreated group. **(E)** Measurement of ICAM-1 in circAFF3-depleted ARPE-19 cells (n = 3). **(F,G)** Immunofluorescence analysis of ICAM-1 following circAFF3 knockdown in ARPE-19 cells. **(F)** Representative images of ICAM-1 (green), with nuclei counterstained by DAPI (blue). Scale bar, 50 μm. **(G)** Quantification of ICAM-1 fluorescence intensity from three independent experiments (n = 3). **(H)** Monocyte adhesion assay after circAFF3 silencing in ARPE-19 cells. Representative images show THP-1 cells labeled with calcein AM attached to ARPE-19 cells labeled with CellTracker™ Red. Scale bar, 200 μm. For quantification, four random fields per sample were captured, and the average number of adherent cells was calculated (n = 12). The expression levels of proinflammatory genes **(C)** and ICAM*-*1 **(E)** were normalized to those of GAPDH. All groups treated with sicircAFF3 were compared with the siCtr group. Data are shown as the mean ± SD. An unpaired two-tailed t-test with Welch’s correction was used for statistical analysis (ns, not significant; *, *p* < 0.05; **, *p* < 0.01; ****, *p* < 0.001).

To investigate the cytoplasmic function of circAFF3, we designed siRNAs to target the back-splicing junction of circAFF3 and transfected ARPE-19 cells to knock down circAFF3 (Figure 2A). The siRNAs targeting circAFF3 effectively reduced circAFF3 levels without affecting the mRNA level of the *AFF3* host gene (Supplementary Figure S2). Given that NF-κB signaling is a key mediator in the inflammatory response of the RPE and is strongly involved in the pathogenesis of dry AMD, we first investigated NF-κB activation after circAFF3 knockdown in ARPE-19 cells using these siRNAs (Almalki and Almujri, 2024). Our experimental results demonstrate that silencing circAFF3 in ARPE-19 cells increased the phosphorylation of p65, a key NF-κB subunit that mediates inflammation in dry AMD (Figure 2B). Consistently, the expression of downstream inflammatory genes, including *ICAM1*, *IL1B*, and *IL6*, was also upregulated under the same conditions (Figure 2C). In contrast, circAFF3 overexpression in ARPE-19 cells decreased the mRNA expressions of *ICAM1*, *IL6*, and *IL1B* (Supplementary Figures S3A,B). Notably, the *Icam1* level was significantly increased when the circAFF3 level was reduced at day 1 post-laser injury (Figure 2D). Therefore, we decided to conduct a more detailed examination of ICAM-1, an adhesion molecule that is upregulated in ARPE-19 cells during inflammation and known to promote monocyte adhesion (Lee et al., 2015). Western blot analysis revealed that circAFF3 knockdown increased total ICAM-1 protein levels, while circAFF3 overexpression decreased ICAM-1 expression in ARPE-19 cells (Figure 2E; Supplementary Figure S3C). Immunofluorescence staining also revealed an elevation in cytoplasmic ICAM-1 in circAFF3-depleted ARPE-19 cells (Figures 2F,G). In addition, monocyte adhesion to ARPE-19 cells was markedly increased after circAFF3 knockdown (Figure 2H). These results demonstrate that a knockdown in circAFF3 expression induces inflammatory responses in ARPE-19 cells by activating NF-κB, increasing the expression of inflammatory genes, and enhancing monocyte adhesion.

**FIGURE 3:**
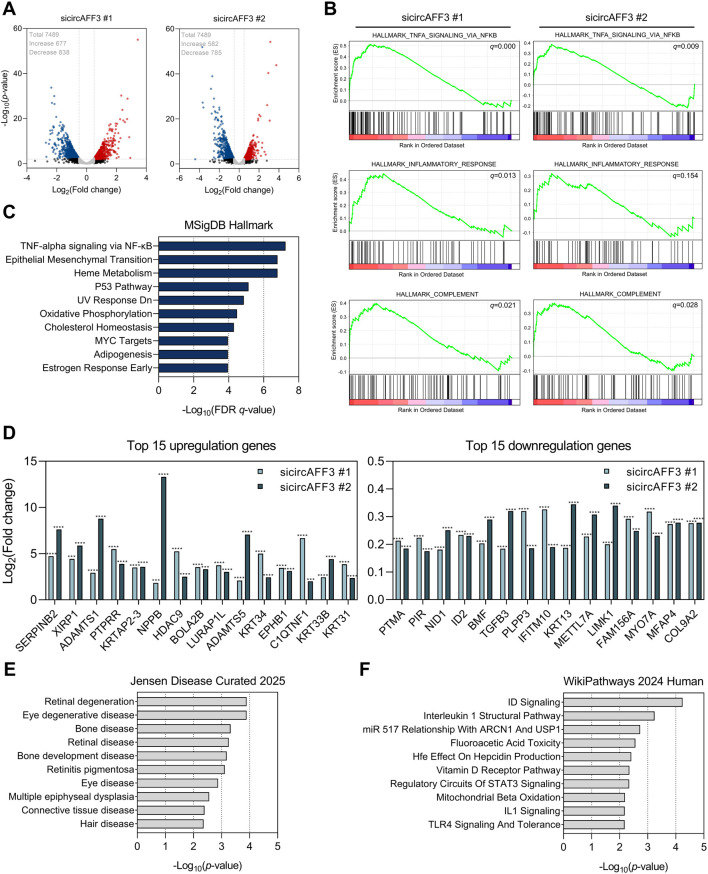
Transcriptome profiling of circAFF3-depleted ARPE-19 cells. **(A)** Volcano plots of the genes with altered expressions in ARPE-19 cells transfected with sicircAFF3 #1 and sicircAFF3 #2. Genes with *p*-values less than 0.05 and an absolute value of log_2_ (fold change) greater than 0.5 are presented as colored dots. **(B)** Gene set enrichment analysis (GSEA) based on 7,071 protein-coding genes with consistent expression changes between sicircAFF3 #1 and #2 treatments and log2 value of counts per million (CPM) ≥ 1. These data indicate significant positive enrichment for gene sets involved in TNF-α signaling via NF-κB, complement activation, and inflammatory response. **(C)** MsigDB hallmark analysis of 409 differentially expressed genes with values of log_2_(CPM) ≥ 4 and |log_2_ (fold change)| ≥ 0.5. The top 10 hallmarks are presented based on the identified false discovery rate (FDR) *q*-value. **(D)** Bar plots of the expression levels of the 15 most up- and downregulated genes from the RNA-seq analysis of circAFF3-depleted ARPE-19 cells. **(E)** Enrichment analysis of downregulated genes using the Jensen Disease Curated 2025 database. **(F)** Pathway enrichment analysis of genes with decreased expression using the WikiPathways 2024 Human database. The top 10 enriched terms in **(E)** and **(F)** are displayed, ranked by statistical significance.

Thus, we subsequently performed mRNA-seq analysis in ARPE-19 cells transfected with each of the two independent sicircAFF3s (#1 and #2) to further determine the molecular mechanism of circAFF3. First, the correlation analysis of the log_2_-transformed fold change in protein-coding genes demonstrated a significant positive correlation between the sicircAFF3 #1- and sicircAFF3 #2-treated groups (r = 0.4025, *p* < 0.0001), indicating consistent expression patterns in both transcriptome sample sets (Supplementary Figure S4). Notably, 7,489 protein-coding genes exhibited the same expression pattern in both groups. Among these, a substantial number of genes were significantly altered after circAFF3 knockdown: 677 genes were upregulated and 838 were downregulated in the sicircAFF3 #1 group; 582 were upregulated and 785 were downregulated in the sicircAFF3 #2 group (Figure 3A). GSEA was then performed, using genes with a log_2_-transformed CPM value of ≥1 for each siRNA. Our results revealed a significant positive NES for gene sets linked to TNF-α signaling via NF-κB, complement activation, and inflammatory response. This reflects the activation of inflammatory pathways after circAFF3 knockdown in ARPE-19 cells (Figure 3B). Additionally, we performed MsigDB hallmark analysis using protein-coding genes with a *p*-value <0.05 and a log_2_(CPM) value ≥4. As a result, we found that genes related to TNF-α signaling via NF-κB signaling were most enriched (Figure 3C). Therefore, the transcriptome analysis confirmed that circAFF3 knockdown in ARPE-19 cells altered the expression of numerous inflammatory genes, particularly those involved in the NF-κB pathway, consistent with our experimental findings.

### Reduction of *ID2* accompanied by elevated oxidative stress and apoptosis upon circAFF3 knockdown

Next, we reviewed the top 30 differentially expressed genes in the RNA-seq analysis. Among these, genes related to RPE function (e.g., *NID1*, *ID2*, and *MYO7A*) were predominantly enriched among those downregulated (Figure 3D) (Semkova et al., 2014; Fan et al., 2018; Gibbs et al., 2003). Therefore, to investigate the impact of this downregulation, we performed enrichment analysis using the Jensen Disease Curated 2025 library in Enrichr. Our studies revealed that circAFF3 knockdown in ARPE-19 cells primarily downregulated genes involved in retinal degeneration (Figure 3E). Subsequently, we analyzed the same subset of genes in the Wikipathways 2024 Human database and confirmed their involvement in the inhibitor of DNA binding (ID) pathway (Figure 3F). These analyses revealed significant changes in genes involved in the ID signaling pathway in ARPE-19 cells following circAFF3 knockdown, suggesting that circAFF3-mediated regulation of ID-related genes may contribute to retinal degeneration.

**FIGURE 4:**
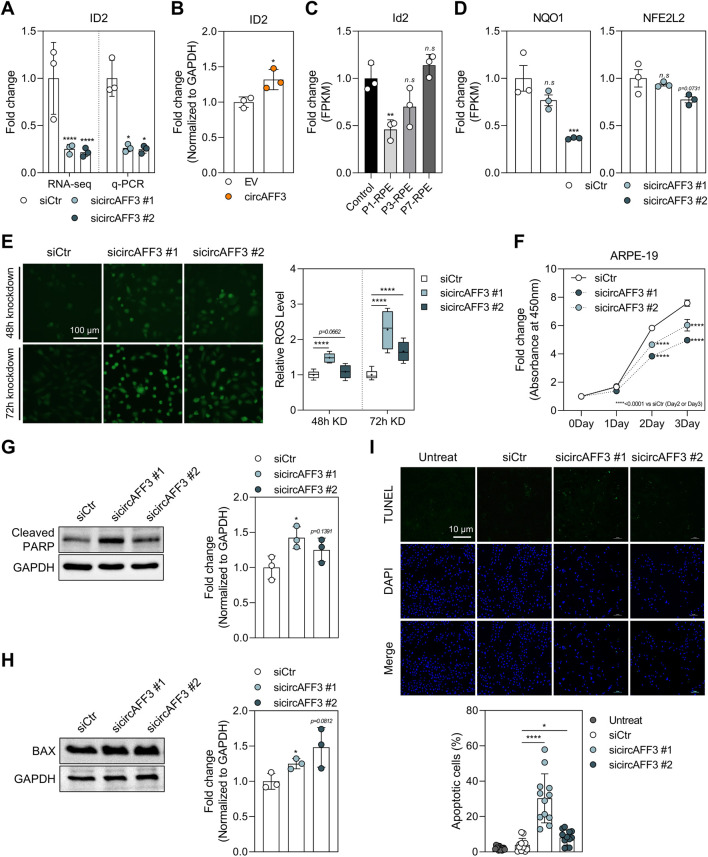
CircAFF3 knockdown–mediated *ID2* repression, leading to oxidative stress and apoptosis. **(A)** qPCR validation of RNA-seq results for *ID2* expression levels in circAFF3-depleted ARPE-19 cells. **(B)** qPCR analysis of *ID2* expression levels after circAFF3 overexpression in ARPE-19 cells. The circAFF3-overexpressing group was compared to the empty vector (EV)-treated control group. **(C)** Fold change of the *ID2* level in the RPE at days 1, 3, and 7 post-laser irradiation (n = 3). The laser-treated group was compared to the untreated group. **(D)** qPCR analysis of the expression change of antioxidant genes (*NQO1*, *NFE2L2*) after circAFF3 knockdown in ARPE-19 cells. **(E)** Measurement of intracellular ROS levels using 2′,7′-dichlorofluorescein diacetate (DCFH-DA) after circAFF3 knockdown in ARPE-19 cells. Representative fluorescence microscopy images (left) show intracellular ROS signals, and quantitative analysis of ROS fluorescence intensity (right) was obtained using a fluorescence microplate reader from the same samples (n = 4, measured in quintuplicate for each). Scale bar, 100 μm. **(F)** Cell viability assessment by the water-soluble tetrazolium (WST) assay after circAFF3 knockdown in ARPE-19 cells. **(G,H)** Western blot analysis of **(G)** cleaved PARP and **(H)** BAX protein expression in circAFF3-depleted ARPE-19 cells (n = 3). **(I)** TUNEL assay to detect the apoptotic circAFF3-depleted ARPE-19 cells. TUNEL-positive cells are indicated in green, with nuclei counterstained by DAPI (blue). Scale bar, 10 μm. The apoptotic index was represented as (TUNEL-positive cells/total DAPI-stained nuclei) × 100 (n = 4, measured in triplicate for each). Expression levels of mRNAs and proteins were normalized to those of GAPDH. All groups treated with sicircAFF3 were compared to the siCtr-treated group. Data are shown as the mean ± SD. An unpaired two-tailed t-test with Welch’s correction was used for statistical analysis (ns, not significant; *, *p* < 0.05; **, *p* < 0.01; ****, *p* < 0.001).

Notably, ID2, a member of the ID protein family, has been reported to function as an antioxidant factor in ARPE-19 cells under oxidative stress, protecting against apoptosis via the p-ERK1/2–ID2–NRF2 signaling pathway (Fan et al., 2018). We found that circAFF3 knockdown reduced *ID2* expression, whereas the *ID2* expression increased in ARPE-19 cells upon circAFF3 overexpression (Figures 4A,B). Consistently, *Id2* expression was also decreased in the RPE samples at days 1 and 3 post-laser injury (Figure 4C). Next, we analyzed the expression of antioxidant-related genes and intracellular ROS levels to further evaluate the impact of circAFF3-mediated *ID2* reduction on antioxidant responses. The *NQO1* and *NFE2L2* gene expression levels were downregulated, which was accompanied by increased ROS accumulation in ARPE-19 cells depleted of circAFF3 (Figures 4D,E). In line with this finding, circAFF3 knockdown significantly inhibited cell survival in both ARPE-19 cells and HRMECs (Figure 4F; Supplementary Figure S5). Western blot analysis also revealed that circAFF3 knockdown enhanced the expression of pro-apoptotic markers, including cleaved PARP and BAX, in ARPE-19 cells (Figures 4G,H). Additionally, apoptotic DNA fragmentation, detected by TUNEL staining, was markedly elevated in circAFF3-depleted cells (Figure 4I). Collectively, these data demonstrate that silencing circAFF3 downregulates the ID2 mRNA level, thereby triggering oxidative stress and apoptosis in ARPE-19 cells.

### Ferroptosis is triggered by circAFF3 knockdown

**FIGURE 5:**
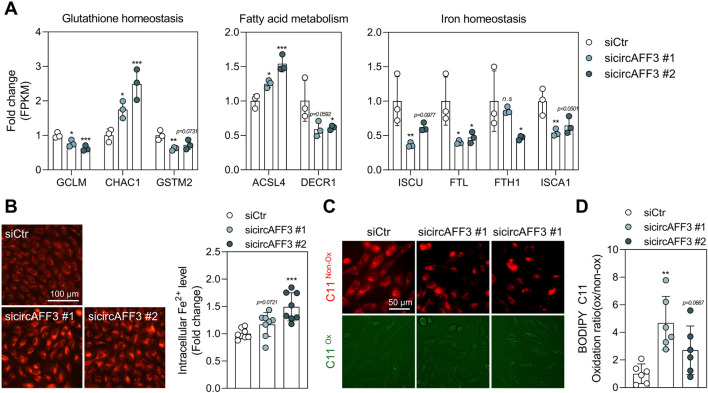
Enhanced ferroptosis phenotypes in circAFF3-depleted ARPE-19 cells. **(A)** Expression changes of ferroptosis-regulating genes as determined by qPCR following circAFF3 knockdown in ARPE-19 cells (n = 3). **(B)** FerroOrange staining to measure the level of labile ferrous ions following circAFF3 knockdown in ARPE-19 cells. Representative images (left) were obtained by fluorescence microscopy, and fluorescence intensity (right) was quantified using the mean gray value in ImageJ software (n = 8). Scale bar, 100 μm. **(C)** Representative fluorescence images of lipid peroxidation as assessed by BODIPY 581/591 C11 staining in circAFF3-depleted ARPE-19 cells. Scale bar, 50 μm. The oxidized form is indicated by green, and the unoxidized form is indicated by red. **(D)** Quantification of lipid peroxidation by fluorescence microplate reader (n = 6), expressed as fold change of oxidized to non-oxidized forms. All sicircAFF3-treated groups were compared with the siCtr group. Data are shown as the mean ± SD. An unpaired two-tailed t-test with Welch’s correction was used for statistical analysis (ns, not significant; *, *p* < 0.05; **, *p* < 0.01; ***, *p* < 0.005).

Ferroptosis, a regulated form of cell death that depends on iron-induced lipid peroxidation, plays a crucial role in the progression of AMD. Indeed, several studies have reported that ferroptotic cell death is also a principal mechanism of RPE loss under oxidative stress, in addition to apoptosis and necrosis (Liu et al., 2024; Totsuka et al., 2019). We observed strong enrichment of ferroptosis-related hallmarks in RNA-seq analysis of ARPE-19 cells depleted of circAFF3 (Figure 3C). Building on these findings, we evaluated whether circAFF3 knockdown promoted ferroptosis. To address this, we examined the expression of genes within ferroptosis-related hallmarks (Supplementary Figure S6). These genes were grouped into glutathione homeostasis, fatty acid metabolism, and iron homeostasis. Significantly, the expression patterns of these genes after circAFF3 knockdown were consistent with the induction of ferroptosis (Figure 5A). Subsequently, we measured two established indicators of ferroptosis: iron accumulation and lipid peroxidation. Accordingly, the FerroOrange assay demonstrated that intracellular iron levels were upregulated, while the BODIPY C11 staining also indicated elevated lipid peroxidation in circAFF3-depleted ARPE-19 cells (Figures 5B–D). These findings show that circAFF3 knockdown also induces ferroptosis in ARPE-19 cells.

Furthermore, our previous results demonstrated that circAFF3 knockdown induced multiple phenotypes, including NF-κB signaling activation, apoptosis, oxidative stress, and ferroptosis. To determine which pathway represents the primary downstream effects of circAFF3 depletion, we examined RNA-seq data from circAFF3-depleted ARPE-19 cells and analyzed gene expression changes within predefined pathway-associated gene sets. Consistent with our prior Hallmark enrichment results, pathway-level ranking based on the average log2 fold change (sicircAFF3 #1 and #2 relative to siCtr) revealed that NF-κB–associated genes exhibited the most transcriptomic alterations, followed by apoptosis-related genes, whereas oxidative stress–and ferroptosis-associated genes showed comparatively moderate changes (Supplementary Table 4; Supplementary Figure S7A). These results indicate that NF-κB activation is the dominant signaling following circAFF3 knockdown. To further investigate whether ferroptosis induction is dependent on NF-κB activation, we performed inhibition experiments using the IKK2 inhibitor TPCA-1. Importantly, the increase in lipid ROS observed in circAFF3-depleted ARPE-19 cells was significantly attenuated in the presence of TPCA-1 (Supplementary Figure S7B). These findings suggest that NF-κB activation functionally contributes to circAFF3 knockdown–induced ferroptosis.

### Regulation of the expression and stability of p53 through direct interaction with circAFF3

**FIGURE 6:**
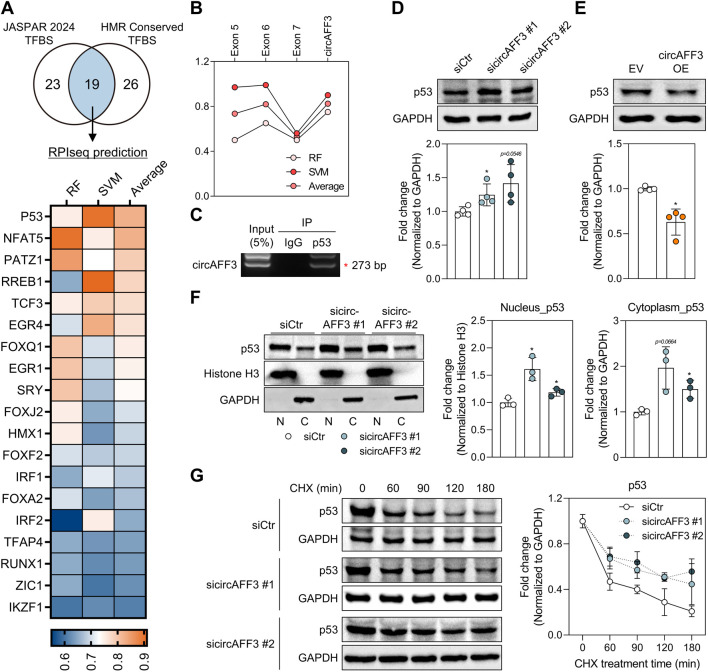
Regulation of p53 expression and stability via direct interaction with circAFF3. **(A)** Selection of candidate transcription factors for *ID2* that are predicted to bind circAFF3. A Venn diagram illustrates transcription factors regulating ID2, identified through the JASPAR database and the HMR Conserved TFBS track in the UCSC Genome Browser. A heatmap shows *ID2* regulatory transcription factors predicted to interact with circAFF3, based on RPIseq analysis. The color bar indicates random forest (RF), support vector machine (SVM), and average (RF + SVM) scores, respectively. **(B)** RPIseq-predicted binding levels of p53 for the full circAFF3 sequence and the included exons 5–7 region. **(C)** Validation of the RNA–protein interactions between circAFF3 and p53 in ARPE-19 cells (n = 3). IP denotes immunoprecipitation. **(D,E)** Western blot analysis of p53 protein expression after circAFF3 **(D)** knockdown or **(E)** overexpression in ARPE-19 cells (n = 3). **(F)** Western blot analysis of the subcellular localization of p53 protein in the circAFF3-depleted ARPE-19 cells (n = 3). Cytoplasmic p53 levels were normalized to GAPDH, and nuclear p53 levels were normalized to Histone H3. **(G)** Cycloheximide (CHX) chase assay measuring p53 protein stability following circAFF3 knockdown in ARPE-19 cells. The circAFF3-overexpressing group was compared with the empty vector (EV)-treated control group. All groups treated with sicircAFF3 were compared to the siCtr-treated group. An unpaired two-tailed t-test with Welch’s correction was used for statistical analysis (ns, not significant; *, *p* < 0.05).

To determine the mechanism through which circAFF3 knockdown regulates ID2 mRNA expression, we first examined transcription factors of *ID2* that might interact with circAFF3. Notably, we identified the transcription factors associated with *ID2* using the JASPAR database and HMR Conserved Transcription Factor Binding Site (TFBS) track in the UCSC Genome Browser. A subsequent analysis using RPIseq predicted that 19 of these associated transcription factors have a high probability of binding to circAFF3 (Figure 6A). Importantly, among these candidates, RPIseq analysis indicated a strong interaction potential between p53 protein and circAFF3, with RF and SVM scores of 0.75 and 0.90, respectively, and identified potential binding sites in exon 6 (Figure 6B). Previously, p53 has been shown to interact directly with a conserved region in the *ID2* promoter, thereby repressing *ID2* expression and inhibiting the proliferation of neural progenitor cells (Paolella et al., 2011). Consistently, the knockdown of *MDM2*, a key regulator of p53 degradation, increased p53 protein expression (Supplementary Figure S8A) (Marine and Lozano, 2010), and this change was accompanied by elevated ID2 mRNA levels and reduced cell viability in ARPE-19 cells (Supplementary Figures S8B,C). This finding led us to focus on p53 as a candidate *ID2* transcriptional regulator that interacts with circAFF3. To experimentally verify this interaction, we conducted a RIP assay in ARPE-19 cells. The results showed that circAFF3 was pulled down in the p53 antibody-immunoprecipitated sample, but not in the IgG-negative control, demonstrating that circAFF3 interacts with p53 in ARPE-19 cells (Figure 6C; Supplementary Figure S9).

Furthermore, circAFF3 knockdown increased p53 protein expression, while circAFF3 overexpression reduced p53 protein levels (Figures 6D,E). However, despite these protein changes, circAFF3 modulation did not affect p53 mRNA expression, suggesting the existence of a post-transcriptional regulatory mechanism (Supplementary Figures S10A,B). In line with this, circAFF3-depleted ARPE-19 cells exhibited enhanced p53 staining, especially in the cytoplasm. Conversely, circAFF3 overexpression resulted in an overall decrease in p53 staining compared to the empty vector-treated group (Supplementary Figures S10C,D). To explore this further, we performed subcellular fractionation, which revealed that p53 protein levels were upregulated in both the nuclear and cytoplasmic fractions from the ARPE-19 cells depleted of circAFF3, indicating that p53 is capable of nucleocytoplasmic shuttling (Figure 6F). In the developing retina, p53 is localized mainly in the nucleus, while MDM2 is predominantly cytoplasmic (Vuong et al., 2012). Meanwhile, MDM2-mediated ubiquitination of p53 occurs in both compartments (Yu et al., 2000). Consistent with this, we observed that MDM2 was primarily localized in the cytoplasm of ARPE-19 cells, and that knockdown of circAFF3 did not alter MDM2 protein levels (Supplementary Figure S11). Overall, these findings suggest that cytoplasmic p53, sequestered by circAFF3, may become more susceptible to degradation by MDM2. Thus, we postulated that depleting circAFF3 facilitates p53 nucleocytoplasmic translocation, ultimately contributing to increased p53 stability. To confirm this hypothesis, we performed a CHX chase assay following circAFF3 knockdown. The stability of p53 was prolonged in the ARPE-19 cells following circAFF3 knockdown compared to the siCtr group (Figure 6G). Consequently, these results indicate that loss of circAFF3 stabilizes the p53 protein by promoting relevant nuclear–cytoplasmic dynamics, resulting in the suppression of ID2 mRNA.

### FOXO3-mediated suppression of *AFF3* and circAFF3 in dry AMD-like conditions

**FIGURE 7:**
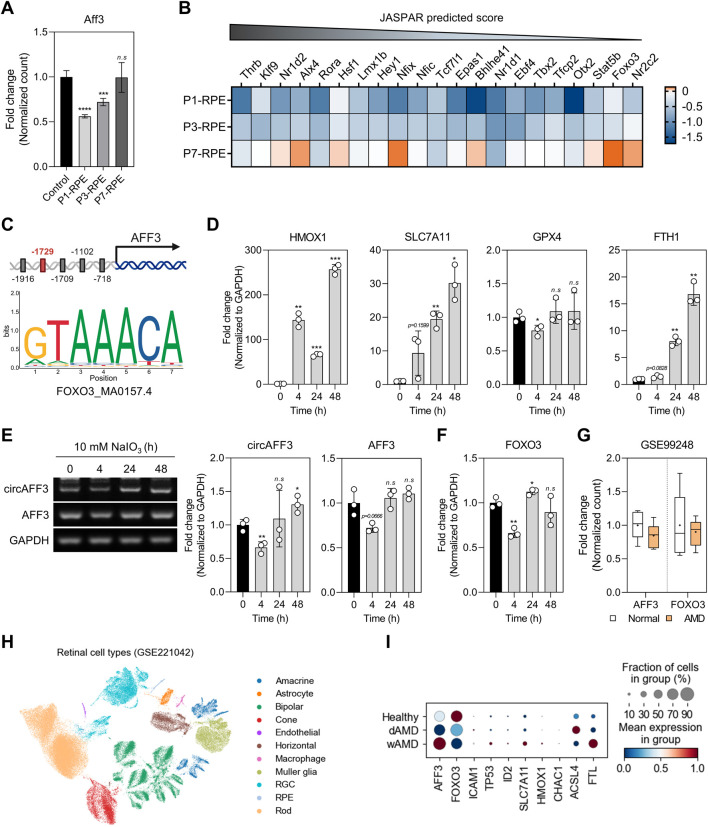
Downregulation of *AFF3* and circAFF3 by FOXO3 under dry AMD-like conditions. **(A)** Fold change in *Aff3* levels in the RPE at days 1, 3, and 7 post-laser irradiation (n = 3). The laser-treated group was compared to the untreated group. **(B)** Heatmap displaying log_2_-transformed fold change of FPKM values for differentially expressed genes in the RPE at day 1 or 3 post-laser injury (n = 3). Genes in the heatmap are ordered according to their JASPAR scores. The color bar represents relative expression compared to the untreated group. **(C)** JASPAR analysis identifying the predicted sequence motif (MA0157.4) of FOXO3 within the promoter region of *AFF3*. Five candidate FOXO3 binding sites were found within the 2000-base-pair upstream promoter region of *AFF3*. Of these, the site located approximately −1729 relative to the transcription start site had the highest JASPAR score and is marked in a red box. **(D)** Expression changes of ferroptosis-related genes at different time points (0, 4, 24, and 48 h) after 10 mM NaIO_3_ treatment in ARPE-19 cells. **(E)** Semi-qPCR analysis of the changes in circAFF3 and *AFF3* levels at different time points (0, 4, 24, and 48 h) after 10 mM NaIO_3_ treatment in ARPE-19 cells (n = 3). **(F)** qPCR analysis of *FOXO3* expression levels at different time points (0, 4, 24, and 48 h) after 10 mM NaIO_3_ treatment in ARPE-19 cells (n = 3). **(G)** Fold change of FPKM values for *AFF3* and *FOXO3* in the RPE of normal (n = 7) and AMD (n = 8) patients, based on the GSE99248 dataset. Expression levels of mRNAs were normalized to those of *GAPDH*. All NaIO_3_-treated groups were compared to the untreated group. Data are shown as the mean ± SD. An unpaired two-tailed t-test with Welch’s correction was used for statistical analysis (ns, not significant; *, *p* < 0.05; ****, *p* < 0.001). **(H)** Uniform manifold approximation and projection (UMAP) visualization of single-nucleus RNA sequencing (snRNA-seq) data from human macular retinal tissues (GSE221042), colored by cell types. **(I)** Dot plot showing the expression levels of *AFF3* and its associated genes in the RPE cell type under different conditions. Color intensity represents the relative average expression level of each gene, and dot size indicates the fraction of RPE cells expressing each gene in each condition.

Next, we examined the expression of the *Aff3* host gene to clarify the mechanism underlying the downregulation of circAff3 in the laser-induced CNV model. We observed a significant reduction in the Aff3 mRNA levels in the RPE samples at days 1 and 3, corresponding to the decrease in circAff3 expression (Figure 7A). This concurrent decrease suggests that reduced *AFF3* transcription may contribute to the downregulation of circAFF3 under pathological conditions. To investigate the upstream regulation, we predicted putative transcription factors for *AFF3* using the JASPAR database. Of these, 21 transcription factors were significantly altered in the RPE samples at day 3 post-laser injury (Figure 7B). Remarkably, FOXO3 has been reported to directly activate target gene expression by binding to the consensus DNA sequences (Morris et al., 2015). Moreover, FOXO3 acts as an antioxidant transcription factor that enhances the expression of antioxidant genes, thereby protecting the RPE cells and photoreceptors from oxidative stress, which suggests that FOXO3 is potentially relevant to AMD pathogenesis (Hao et al., 2024). Given this evidence, FOXO3 was prioritized as a candidate transcription factor regulating *AFF3* in the pathology of AMD. The subsequent JASPAR analysis predicted a specific FOXO3-binding motif in the *AFF3* promoter (Figure 7C). Then, we examined whether human circAFF3, *AFF3*, and *FOXO3* exhibit consistent reductions in expression under dry AMD-like conditions. Accordingly, we used ARPE-19 cells treated with NaIO_3,_ a well-known oxidative stress inducer that mimics dry AMD. NaIO_3_-induced RPE cell death was ferroptosis-dependent (Liu et al., 2021), and the expressions of known ferroptosis markers (*HMOX1*, *SLC7A11*, *GPX4*, and *FTH1*) were modulated at 4, 24, and 48 h after treatment (Figure 7D). A similar expression change was observed in the RPE samples at day 3 post-laser injury (Supplementary Figure S12). In the NaIO_3_-induced ARPE-19 cells, the expression of circAFF3, *AFF3*, and *FOXO3* was significantly reduced at 4 h after treatment, with levels recovering later (Figures 7E,F). Analysis of the public GEO dataset (GSE99248) revealed parallel changes in AFF3 and FOXO3 expression in RPE from AMD patients (Figure 7G). Together, these data highlight that FOXO3-mediated reduction of *AFF3* is a key mechanism for the downregulation of circAFF3 under dry AMD-like conditions.

To determine whether these mechanisms are recapitulated *in vivo*, we analyzed publicly available single-nucleus RNA sequencing (snRNA-seq) data from macular lesions of AMD patients and control donors (GSE221042). We first confirmed cell-type annotation using established retinal markers and examined *AFF3* expression across retinal cell populations (Figure 7H; Supplementary Figures S13A,B). *AFF3* expression was enriched in neuronal cell types, including amacrine, horizontal, and RGC cells, and was also detected in a substantial fraction of RPE cells (Supplementary Figure S13C). Notably, in RPE cells, *AFF3* expression was decreased in dry AMD samples and increased in wet AMD samples compared with healthy controls, consistent with the expression pattern observed in the laser-induced CNV model (Supplementary Figure S13D). We next examined the expression patterns of genes associated with the proposed downstream pathways of *AFF3*, including *TP53*, *ID2*, *ICAM1*, and ferroptosis-related markers (*SLC7A11*, *HMOX1*, *CHAC1*, *ACSL4*, and *FTL*), as well as the upstream transcriptional regulator *FOXO3*. In dry AMD samples, these genes showed transcriptional patterns similar to those observed in circAFF3-depleted ARPE-19 cells and the laser-induced CNV model (Figure 7I). Collectively, these data support that our findings in ARPE-19 cells are also relevant in human dry AMD and suggest a potential link to disease-associated mechanisms.

## Discussion

**FIGURE 8:**
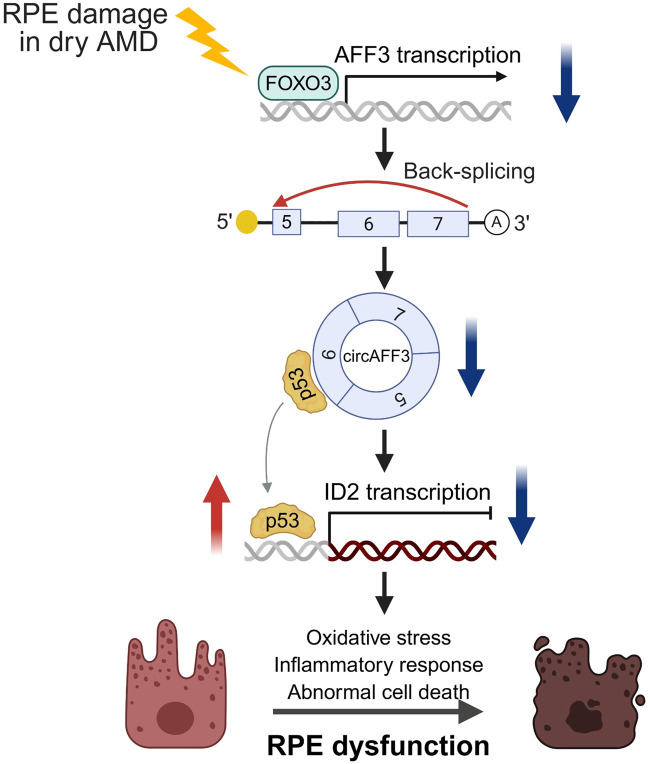
Schematic overview of the role of the circAFF3 in regulating RPE degeneration. In dry AMD, circAFF3 is reduced in RPE cells. Loss of circAFF3 promotes nuclear accumulation of p53, resulting in *ID2* suppression. This pathway leads to oxidative stress, activation of p65-mediated inflammation, and abnormal cell death, ultimately contributing to RPE degeneration.

This study demonstrated that circAFF3 expression is consistently downregulated in models of dry AMD pathogenesis, including RPE at day 3 post-laser injury and NaIO_3_-treated ARPE-19 cells. Functional analysis of circAFF3 revealed that silencing circAFF3 triggers inflammatory responses through p65 activation in ARPE-19 cells. Additionally, we demonstrated that circAFF3, which is predominantly localized in the cytoplasm, directly binds to p53 in ARPE-19 cells. Accordingly, depletion of circAFF3 facilitated the translocation of p53 between the nucleus and cytoplasm, leading to the nuclear accumulation of p53. Subsequently, elevated nuclear p53 repressed *ID2* expression, promoting oxidative stress and inducing regulated cell death pathways, such as apoptosis and ferroptosis. In summary, these results reveal that circAFF3 controls oxidative stress and cell fate by modulating a circAFF3/p53/*ID2* pathway (Figure 8). Given that RPE dysfunction is a core feature of dry AMD pathogenesis, our findings suggest that circAFF3 may be a promising therapeutic target for dry AMD.

Previous studies have shown that oxidative stress in RPE cells induces an inflammatory microenvironment, including NF-κB-dependent cytokine production, thereby implicating AMD pathogenesis (Tan et al., 2020; Wu et al., 2010). Chronic oxidative stress and inflammatory signaling contribute to RPE dysfunction and degeneration, which can progress to RPE cell death during advanced stages of AMD (Datta et al., 2017). In our results, considering these phenotypic time points, circAFF3 knockdown induced ROS accumulation and NF-κB activation at 48 h, and a significant increase in cell death was observed at 72 h (Figures 2, 4). These temporal patterns support that oxidative stress and inflammatory signaling may precede and potentially contribute to progressive cell death in ARPE-19 cells. Collectively, these observations suggest that the circAFF3/p53/ID2 axis functions as an upstream regulatory mechanism that triggers oxidative stress, thereby facilitating secondary inflammatory activation and contributing to RPE cell death.

In addition, this study shows that circAFF3 knockdown significantly increased ICAM-1 expression and monocyte adhesion to ARPE-19 cells (Figures 2C–H). ICAM-1 is a transmembrane glycoprotein that mediates leukocyte attachment by interacting with β2 integrins, including LFA-1 and Mac-1 (Bui et al., 2020). In ARPE-19 cells, inflammatory stimuli such as IL-1β and TNF-α upregulate ICAM-1 expression, which promotes monocyte adhesion (Huang et al., 2020; Yang et al., 2016). Given that subretinal leukocyte accumulation and chronic inflammation are key features of AMD pathogenesis (Tan et al., 2020), increased ICAM-1 following circAFF3 knockdown may reflect an inflammatory activation state relevant to dry AMD progression. Additionally, circAFF3 knockdown increased ICAM-1 levels at both the cell membrane and within the cytoplasm (Figures 2F,G). ICAM-1 induced by inflammatory stimuli is distributed throughout the whole cells and undergoes intracellular trafficking through the endoplasmic reticulum and Golgi apparatus before reaching the cell membrane in A549 (Mitsuda et al., 2014). Therefore, the increased cytoplasmic ICAM-1 following circAFF3 depletion may represent an intracellular trafficking intermediate toward surface expression.

In the process of selecting circRNA candidates associated with dry AMD, we identified 18 significantly altered circRNAs in the RPE sample from the laser-induced CNV model at day 3 (Figure 1A). Among them, circARHGAP10 has been implicated in myotonic dystrophy type 1 through co-regulation of miR-409-3p, which affects DMPK levels, RNA foci, and splicing defects (Baci et al., 2025). Meanwhile, circARHGAP10 has also been reported to promote the osteogenic differentiation of valvular interstitial cells via the miR-355-3p/*RUNX2* axis (Jiang et al., 2023). Additionally, circRNF169 accelerates senescence of mouse embryonic fibroblasts by sponging miR-30c-5p (Zhang et al., 2022). Notably, since the functions of circARHGAP10 and circRNF169 have already been characterized in non-ocular diseases, we excluded these circRNAs from additional validation in our study. However, the potential roles of these circRNAs in ocular disease mechanisms remain unclear and require further studies.

Our study demonstrated that circAFF3 knockdown activates p65, resulting in increased expression of inflammatory genes and subsequent monocyte recruitment in ARPE-19 cells (Figure 2). Upon cellular stress, p65, a key member of the NF-κB family, translocates into the nucleus and activates genes that regulate inflammation, immune responses, cell survival, and angiogenesis (Almalki and Almujri, 2024). Importantly, the angiogenic role of NF-κB signaling, which induces angiogenic factors, such as VEGF, contributes to the pathological neovascularization of wet AMD (Lim et al., 2012). These findings suggest a potential role for circAFF3 in modulating angiogenic processes in wet AMD by regulating NF-κB activity.

Additionally, the RNA-seq analysis of the circAFF3-depleted ARPE-19 cells presented the highest number of differentially regulated genes (Figure 3D). Among these, *NID1* was significantly downregulated following the knockdown of circAFF3 in ARPE-19 cells. The *NID1* gene encodes nidogen-1, a basement membrane protein that connects laminin and type IV collagen, contributing to the structural integrity of BrM (Timpl and Brown, 1996). Thus, a loss of integrity in the BrM is well established as a prerequisite for CNV (Chong et al., 2005). Consistently, previous studies have demonstrated that disruption of the BrM in *Nid1* knockout mice increases the CNV area relative to wild-type controls (Semkova et al., 2014). In light of these reports, we propose that circAFF3 knockdown can induce a reduction in *NID1* expression in ARPE-19 cells, which may impair BrM stability and thereby create conditions that facilitate neovascularization in AMD.

We also revealed that circAFF3 knockdown led to both increased p53 expression and activation of p65 in ARPE-19 cells (Figures 2B, 6D). Although this study did not establish a causal relationship between these two observations, a prior study revealed that p53 can activate NF-κB via a nonclassical RSK1-dependent pathway involving p65 phosphorylation, thereby contributing to apoptosis (Bohuslav et al., 2004). Furthermore, p53 and NF-κB also act cooperatively to promote the expression of proinflammatory cytokine and chemokine genes in human macrophages (Lowe et al., 2014). This evidence suggests that the p53–NF-κB signaling cascade contributes to both inflammatory and apoptotic responses.

Moreover, we confirmed that circAFF3 knockdown can induce both apoptosis and ferroptosis in ARPE-19 cells (Figures 4, 5). Ferroptosis is an iron-dependent form of cell death marked by lipid peroxidation and plasma membrane rupture (Dixon et al., 2012). Iron is a biologically essential element that requires strict regulation of intracellular levels in the retina (Song and Dunaief, 2013). Importantly, increased iron levels are observed in the macula and aqueous humor of patients with AMD compared to controls, suggesting that iron accumulation may contribute to the disease (Hahn et al., 2003; Jünemann et al., 2013). Thus, ferroptosis is now also recognized as a critical pathogenic mechanism in the progression of AMD (Liu et al., 2024; Totsuka et al., 2019; Liu et al., 2021). Additionally, glutathione, a major cellular antioxidant, detoxifies lipid peroxides through GPX4, while the depletion of glutathione induces ferroptosis in the RPE (Sun et al., 2018). Consistently, our study identified genes involved in the homeostasis of glutathione, fatty acids, and iron with altered expressions, suggesting activation of ferroptosis (Figure 5A). Specifically, changes in the expression of genes related to glutathione metabolism (*GCLM*, *CHAC1*, and *GSTM2*) reflect disturbances in antioxidant defense mechanisms (Sun et al., 2018; Wang et al., 2022a; Sun et al., 2024; Xiong et al., 2025). The dysregulation of genes associated with fatty acid metabolism (*ACSL4* and *DECR1*) indicated increased susceptibility to lipid peroxidation (Doll et al., 2017; Yang et al., 2025). Lastly, changes in iron-related genes (*FTL*, *FTH1*, *ISCA1*, and *ISCU*) suggested impaired iron storage and Fe–S cluster biogenesis (Tang et al., 2021; Du et al., 2023).

Furthermore, regulated cell death in the RPE is a core pathological feature of AMD, thereby making the elucidation of these mechanisms essential (Zhang et al., 2025). While previous studies regarded apoptosis as the main pathway in the death of RPE cells (Adler et al., 1999), new evidence suggests other types of cell death-related pathways, including necroptosis, pyroptosis, and ferroptosis, may also contribute to AMD pathology (Hanus et al., 2015; Zhou et al., 2023; Liu et al., 2024). Thus, AMD progression likely does not involve a single form of cell death, but rather an interplay among multiple pathways. Nonetheless, further investigations are needed to determine whether circAFF3 knockdown engages additional cell death pathways.

*FOXO3* encodes the transcription factor forkhead box O-3, a longevity-associated gene that regulates cellular stress resistance against age-related diseases (Morris et al., 2015). Several studies have shown that dysfunction of FOXO3a is closely related to age-related ocular diseases, including AMD, cataracts, and glaucoma (Hao et al., 2024). Indeed, acetylated FOXO3 in ARPE-19 cells under oxidative stress binds to the *CFH* promoter, thereby preventing interaction with STAT1 and reducing *CFH* expression. This effect downregulates the complementary pathway, potentially increasing the risk of AMD (Wu et al., 2007). Additionally, vitamin C protects ARPE-19 cells from oxidative stress by upregulating *p53* and *FOXO3* expression in a SIRT1-dependent manner, a key regulator of the stress response (Wei et al., 2014). Beyond ocular diseases, FOXO3 has also been shown to be inversely regulated by ferroptosis, suggesting that FOXO3 may be a potential therapeutic target for modulating ferroptosis in osteoarthritis (Zhao et al., 2023).

Our study elucidated the function of circAFF3, which is expressed specifically in the RPE but is rarely expressed in HRMEC and HUVEC (Figure 1D). Notably, circRNAs have been investigated as promising biomarkers for various diseases due to their structural stability, high specificity, and presence in body fluids (e.g., blood, urine, cerebrospinal fluid) (Verduci et al., 2021). For example, in retinal diseases, circ_0005015 is upregulated in the vitreous, plasma, and pre-retinal fibrovascular membranes (FVMs) of diabetic retinopathy (DR) patients, highlighting circ_0005015 as a potential biomarker for determining the prognosis, diagnosis, or treatment of DR (Zhang et al., 2017). However, although the RPE-specific circAFF3 shows potential as a therapeutic target, the utility of circAFF3 as a non-invasive biomarker is limited due to the challenges associated with acquiring samples directly from the RPE.

Several studies have demonstrated that circRNAs are stably present in extracellular vesicles (EVs) in human bodily fluids, and exosomal circRNAs have been proposed as biomarkers in cancer research for both diagnosis and prognosis (Zhao et al., 2024; Wang et al., 2019). The polarized RPE secretes EVs, including exosomes, in both apical and basolateral directions (Klingeborn et al., 2017). Hence, tear fluid, which can be collected non-invasively, contains abundant EVs, making it an attractive biofluid for ocular biomarker research (Phan et al., 2025; Fatima et al., 2025). Although the presence of EVs in tears has previously been established, direct evidence that RPE-derived EVs reach the tear film requires further investigation. Nevertheless, the secretion of EVs from the RPE into adjacent compartments supports the potential detection of EVs in tears (Flores-Bellver et al., 2021; Lee et al., 2024). Thus, if methods to identify RPE-derived EVs in tears are developed, circAFF3 could serve as a promising non-invasive biomarker for diagnosing and predicting dry AMD (Su et al., 2024).

## Data Availability

The original contributions presented in the study are included in the article/Supplementary Material, further inquiries can be directed to the corresponding author.
